# Feasibility and safety of hepatic artery infusion chemotherapy via the distal transradial access for hepatocellular carcinoma

**DOI:** 10.3389/fonc.2025.1644296

**Published:** 2025-10-22

**Authors:** Yi Wu, Chunlin Yu, Xinghai Li, Baoliang Zhong, Daolin Zeng, Yunfei Tian

**Affiliations:** Interventional Radiology, The People’s Hospital of Ganzhou, Ganzhou, Jiangxi, China

**Keywords:** hepatocellular carcinoma, distal transradial access (dTRA), hepaticartery infusion chemotherapy, feasibility, safety

## Abstract

**Background and Purpose:**

Hepatocellular carcinoma (HCC) remains a global health challenge, with hepatic artery infusion chemotherapy (HAIC) serving as a pivotal treatment for unresectable cases. Traditional transfemoral access (TFA) for HAIC is associated with significant limitations, including prolonged immobility, increased risk of deep vein thrombosis (DVT), and reduced quality of life. The distal transradial access (dTRA), emerging as a viable alternative in coronary and neurovascular interventions, offers potential advantages in HAIC. This study evaluates the feasibility, safety, and clinical outcomes of dTRA for HAIC in HCC patients, addressing the paucity of data in this specific application.

**Methods:**

A retrospective analysis was conducted on patients who underwent dTRA-HAIC procedures for HCC between November 2023 and December 2024. The puncture time, procedural time, incidence of distal radial artery occlusion (d-RAO) and access site complications (ASC) were used to evaluate the treatment efficacy in the patients. Univariate and multivariate logistic regression analysis was performed to identify predictive factors associated with d-RAO development.

**Results:**

The mean puncture time was 3.5 minutes (3–4.25 minutes), with a distal radial artery diameter of 1.96 ± 0.16 mm. The mean catheter indwelling time for mFOLFOX-HAIC and Ralox-HAIC were 2375 minutes (1715–3276 minutes) and 410 minutes (276, 544 minutes), respectively. Early d-RAO rates were 25.4% on postoperative day 1, declining to 20.6% at 6 months. Multivariate analysis identified preoperative D-dimer levels (p < 0.05) as significant risk factors for early d-RAO in one-month after operation. Multiple punctures may be associated with a high risk of d-RAO. No severe bleeding, hematoma, or pseudoaneurysm occurred.

**Conclusion:**

dTRA for HAIC demonstrates high technical feasibility and acceptable safety, representing a promising alternative to TFA. Preoperative D-dimer screening and limitations on repeated punctures may optimize outcomes. Larger multicenter studies are warranted to validate these findings and refine patient selection criteria.

## 1 Introduction

Hepatocellular carcinoma (HCC) ranks as the sixth most common cancer globally and the third leading cause of cancer-related death, with over 90% of cases occurring in developing countries, including China (1). Especially China, with a high incidence of liver cancer, more than one-half of patients presented with stage III-IV disease (56.9%, *n* = 1702), precluding curative resection (2). Hepatic artery infusion chemotherapy (HAIC) has emerged as a critical treatment modality for unresectable HCC, delivering chemotherapeutic agents directly to tumor vasculature while minimizing systemic toxicity (3–6). The commonly used HAIC include mFOLFOX-HAIC (4, 7, 8), with continuous perfusion for 26–46 hours, and Ralox-HAIC, generally perfusion for 4–6 hours (9). Traditional HAIC is typically performed via the transfemoral approach (TFA), which mandates prolonged bed rest (6–12 hours), increases deep vein thrombosis (DVT) risk, and imposes significant discomfort due to groin compression (10).These limitations underscore the need for minimally invasive alternatives that preserve patient mobility and reduce vascular complications.

The transradial access (TRA) route, initially described for coronary interventions, has gained prominence for its lower bleeding risk and improved patient comfort compared to TFA (11). Distal transradial access (dTRA), targeting the radial artery at the anatomical snuffbox (distal to the superficial palmar arch), offers distinct advantages over traditional wrist-level TRA. Anatomically, the distal radial artery lies superficial to the scaphoid and trapezium bones, facilitating easier hemostasis and reducing compression time (12). Physiologically, preserving flow through the superficial palmar arch minimizes the risk of forearm radial artery occlusion (RAO), a critical concern for repeat interventions (13).

While dTRA has been validated in coronary angiography and transarterial chemoembolization (TACE) (14–16),its application in HAIC remains underinvestigated. Early studies in TACE demonstrate comparable technical success rates (86.2%-95.13%) and low major complication rates (0%-5.7%) with dTRA, highlighting its potential for hepatic interventions (17, 18). The anatomical snuffbox puncture site avoids the hypothenar motor branch and provides a straight trajectory to the aortic arch, potentially simplifying catheter navigation to the hepatic artery (19).

Despite these advancements, HAIC-specific data on dTRA are scarce. Technical feasibility, safety profiles, particularly d-RAO rates and impact of repeated punctures on radial artery patency in long-duration procedures requiring prolonged catheter retention are still key uncertainties. This study aims to address these gaps by evaluating dTRA-HAIC in a real-world HCC cohort, focusing on procedural outcomes, complication rates, and predictors of d-RAO.

## 2 Methods

### 2.1 Study design and patient selection

This single-center retrospective cohort study included 44 patients who underwent 63 dTRA-HAIC procedures for hepatocellular carcinoma between November 2023 and December 2024. The study was approved by the institutional ethics committee (approval no. PJB2025-225-01), with informed consent waived due to its retrospective nature. In this study, important measures such as data anonymization and data access control were adopted to ensure the privacy and security of patients.

Inclusion criteria: (1) Patients with hepatocellular carcinoma (HCC) confirmed by pathology or imaging; (2) underwent hepatic artery infusion chemotherapy (HAIC) via the distal transradial access (d-TRA); (3) with complete clinical data. Exclusion criteria included: (1) concurrent severe comorbidities (e.g., severe cardiac dysfunction, renal failure); (2) preexisting occlusion or significant vascular abnormalities of the distal radial artery; (3) intraoperative conversion to alternative access routes (e.g., femoral artery approach).

### 2.2 Procedural technique

#### 2.2.1 Puncture and sheath placement

**Figure 1:**
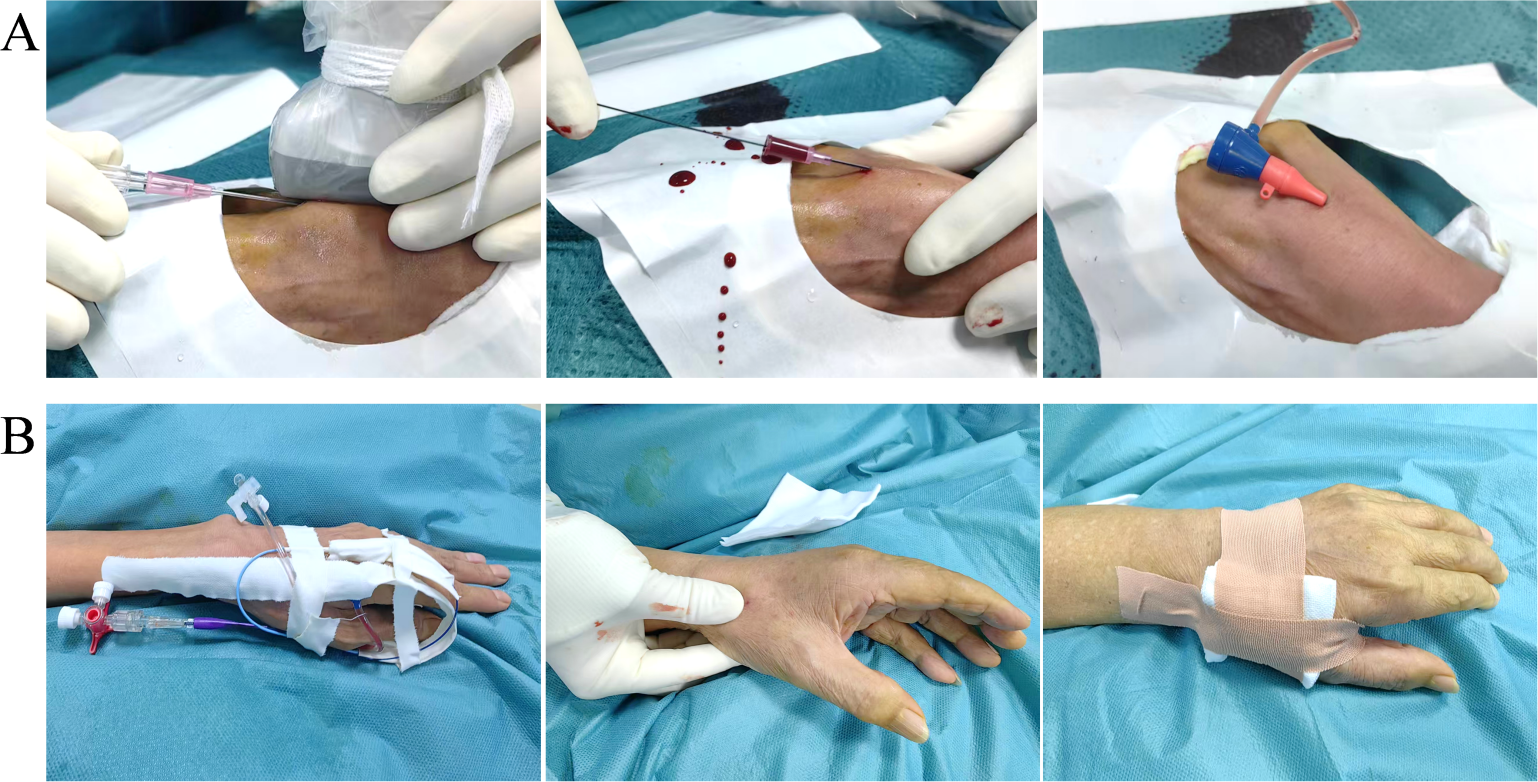
Technical procedure. **(A)** Transradial artery puncture, guidewire insertion and 4F radial sheath placement. **(B)** Catheter fixation, sheath removal, and compression with elastic bandage.

Patients were positioned supine with the punctured hand resting on the lower abdomen. Under Doppler ultrasound guidance (SonoScape S70N), the distal radial artery at the anatomical snuffbox was punctured using a 21G needle (Merit Medical). Following successful blood return, a 0.018-inch guidewire was advanced, and a 4F microsheath (Merit Medical) or 4F radial sheath (APT) was deployed (**Figure 1A**). The sheath was flushed with a cocktail of nitroglycerin (100 μg), lidocaine (1 mL), and heparin (2000 U) to minimize spasm and thrombosis.

#### 2.2.2 Catheter navigation and HAIC

A 125-cm MPA1/Ultimate1 catheter (Cordis) and 180-cm 0.035-inch guidewire (Terumo) were used to navigate through the brachial, axillary, and subclavian arteries to the hepatic artery. Angiography confirmed tumor-feeding vessels before infusing chemotherapy (mFOLFOX: oxaliplatin 85 mg/m^2^ intra-arterial infusion on day 1, levoleucovorin Calcium 200 mg/m^2^ intra-arterial infusion on day 1, and 5-fluorouracil, 400 mg/m^2^ bolus infusion on day 1 and 2400 mg/m^2^ continuous infusion over 26 or 46 h, or Ralox: oxaliplatin 100 mg/m^2^ for 3∼5 h, raltitrexed 3 mg/m^2^ in 1 h). Post-procedure, the sheath was removed, and manual compression with an elastic bandage was applied for 3 hours (**Figure 1B**).

### 2.3 Data collection and outcomes

The following data were collected: patient age, gender, tumor stages, tumor sizes, underlying medical conditions (e.g. liver cirrhosis, hypertension, diabetes mellitus, tobacco smoking, alcohol consumption, anticoagulants), procedural details of TRA (including access kit, puncture time, diameter of the distal radial artery and procedural time), incidence of distal radial artery occlusion (d-RAO) and vascular recanalization after the procedure, the preoperative baseline indicators, such as platelet count, prothrombin time (PT) and D-dimer, total cholesterol and so on just before the procedure.

The main observation indicators included: (1) the puncture time, diameter of the distal radial artery and procedural time; (2) incidence of distal radial artery occlusion (d-RAO), defined as the absence of radial artery flow on postoperative ultrasound; (3) access sites complications (bleeding, hematoma, arterial dissection, pseudoaneurysm, etc.); (4) vascular recanalization. Univariate analysis was performed to identify predictive factors associated with d-RAO development.

### 2.4 Statistical analysis

Categorical variables were presented as frequencies and percentages, while continuous variables were expressed as medians [interquartile range] or means ± standard deviation. Group comparisons were performed using the χ² test or Mann-Whitney U test. Preoperative variables were analyzed for associations with d-RAO using univariate and multivariate logistic regression analysis. All analyses were conducted using SPSS 26.0, with *p* < 0.05 considered statistically significant.

## 3 Results

### 3.1 Baseline characteristics

Forty-four patients (79.5% male) with a mean age of 57 years (range 47–61) were included. Most had advanced HCC (CNLC IIIA: 40.9%, IIIB: 29.5%), large tumors (>5 cm: 65.9%), and underlying cirrhosis (84.1%). Comorbidities included hypertension (54.5%) and diabetes (31.8%). Baseline demographics are detailed in **Table 1**.

**Table 1:** Clinical characteristics of patients.

Clinical characteristics of patients (N = 44)		
Age, years		57 (47, 61)
Gender	Male	35 (79.55%)
	Femal	9 (20.45%)
Clinical stage, n (%)	IA	4 (9.09%)
	IB	3 (6.82%)
	IIA	3 (6.82%)
	IIB	3 (6.82%)
	IIIA	18 (40.91%)
	IIIB	13 (29.55%)
Tumor diameter, n (%)	<30mm	12 (27.27%)
	30 - 50mm	3 (6.82%)
	50 - 100mm	11 (25.00%)
	>100mm	18 (40.91%)
Liver cirrhosis	Yes	37 (84.09%)
	No	7 (15.91%)
History of other diseases	Coronary heart disease	1 (2.27%)
	Heart failure and atrial fibrillation	1 (2.27%)
	None	42 (95.45%)
Smoking	yes	15 (34.09%)
	no	29 (65.91%)
Drinking alcohol	Yes	11 (25.00%)
	no	33 (75.00%)

### 3.2 Procedural outcomes

A total of 63 procedures were performed (left dTRA: 73.0%, right dTRA: 27.0%). Technical success (defined as successful hepatic artery cannulation) was achieved in all cases. Median puncture time was 3.5 minutes (IQR 3–4.25), with a mean distal radial artery diameter of 1.96 ± 0.16 mm. The mean catheter indwelling time for mFOLFOX-HAIC and Ralox-HAIC was 2375 minutes (IQR 1715–3276 minutes) and 410 minutes (IQR 276, 544 minutes), respectively. Eighteen patients underwent repeated punctures (2–4 times), with one patient requiring four sessions (**Table 2**).

**Table 2:** Procedural details of dTRA.

Procedural details of dTRA(N = 63)		
Vascular sheath (4F microsheath/4F radial sheath)		29/34
Puncture time (min), median[range]		3.5 [3, 4.25]
Left/Right		46/17
Cocktails (yes/no)		40/13
Anticoagulation during catheterization (yes/no)		18/45
Diameter of the distal radial artery (mm), mean ± SD		1.96 ± 0.16
Duration of the operation (min), median[range]		29 [20.5, 35.5]
Catheter indwelling time (min), median[range]	mFOLFOX-HAIC (n=35)	2375 [1715, 3276]
	Ralox-HAIC (n=28)	410 [276, 544]

dTRA, distal transradial access; SD, standard deviation; HAIC, hepatic artery infusion chemotherapy.

### 3.3 Safety and complications

#### 3.3.1 Distal radial artery occlusion

The d-RAO incidence decreased over time: 25.4% (16/63) on day 1, 22.2% (14/63) at 1 month, 19.1% (12/63) at 3 months, and 20.6% (13/63) at 6 months (**Table 3**). Vascular recanalization occurred in 4 cases (6.3%): 2 within 1 month and 2 between 1–3 months. No recanalization was observed after 3 months (**Table 4**).

**Table 3:** The d-RAO of patients undergoing dTRA during the follow-up period.

The d-RAO of patients undergoing dTRA during the follow-up period		
Follow-up period	n	%
On the following day	16	25.40%
One-month	14	22.22%
3-month	12	19.05%
6-month	13	20.63%

d-RAO, distal radial artery occlusion; dTRA, distal transradial access.

**Table 4:** Vascular recanalization following d-RAO in patients undergoing dTRA.

Vascular recanalization following d-RAO in patients undergoing dTRA	
Period after the operation	Vascular recanalization following d-RAO, n
From the following day to one month	2
From one month to 3 months	2
From 3 months to 6 months	0

d-RAO, distal radial artery occlusion; dTRA, distal transradial access.

#### 3.3.2 Risk Factors for d-RAO

Univariate analysis revealed that D-dimer was significantly associated with an increased risk of d-RAO (OR = 1.35, 95% CI: 1.08-1.69, p = 0.009) (**Table 5**). After adjusting for potential confounders (including smoking, operation time, catheter indwelling time and so on), multivariate analysis demonstrated that D-dimer remained an independent risk factor for d-RAO (adjusted OR = 1.29, 95% CI: 1.03-1.62, p = 0.029) (**Table 5**). This suggests that D-dimer provides independent prognostic value for predicting d-RAO in the first post-operation month. Notably, the sole patient with four punctures developed persistent d-RAO at all time points (**Table 6**).

**Table 5:** The influence of preoperative baseline indicators on d-RAO following dTRA.

Univariate and multivariate logistic regression analyses of baseline variables predicting d-RAO in one-month later						
Factors	Univariate			Multivariate		
	OR	95%CI	P	OR	95%CI	P
Gender: Male/Female	0.85	0.16 ~ 4.57	0.854			
Age	0.99	0.94 ~ 1.04	0.708			
Smoking	2.51	0.75 ~ 8.42	0.136			
Dringking	1.41	0.42 ~ 4.74	0.577			
Cirrhosis	0.42	0.10 ~ 1.70	0.223			
dTRA: left/right	0.90	0.24 ~ 3.38	0.879			
Cocktails	1.74	0.34 ~ 8.96	0.510			
Anticoagulation during catheterization	1.00	0.27 ~ 3.72	1.000			
HAIC: mFOLFOX/Ralox	0.63	0.18 ~ 2.15	0.458			
Duration of the operation	1.00	1.00 ~ 1.00	0.860			
Puncture time	1.38	0.93 ~ 2.06	0.109			
Diameter of the distal radial artery	0.23	0.01 ~ 9.75	0.442			
Catheter indwelling time	1.00	1.00 ~ 1.00	0.367			
Blood platelet	1.00	1.00 ~ 1.01	0.627			
Serum creatinine	1.00	0.96 ~ 1.04	0.957			
Total cholesterol	1.44	0.92 ~ 2.24	0.107			
Triglyceride	0.89	0.44 ~ 1.79	0.742			
HDL-C	4.26	1.01 ~ 17.99	**0.048**	2.73	0.65 ~11.37	0.169
LDL-C	1.14	0.90 ~ 1.46	0.272			
PT	0.96	0.68 ~ 1.35	0.821			
D-dimer	1.35	1.08 ~ 1.69	**0.009**	1.29	1.03 ~ 1.62	**0.029**
Total bilirubin	1.04	0.98 ~ 1.09	0.166			
Albumin	1.01	0.92 ~ 1.12	0.805			

d-RAO, distal radial artery occlusion; dTRA, distal transradial access; HAIC, hepatic artery infusion chemotherapy; HDL-C, high density lipoprotein cholesterol; LDL-C, low density lipoprotein cholesterin; PT, prothrombin time; OR, Odds Ratio; CI, Confidence Interval. Bold values are values with statistical differences (P<0.05).

**Table 6:** The number of punctures affected on d-RAO in patients undergoing dTRA.

The number of punctures affected on d-RAO in patients undergoing dTRA				
Number of punctures	Distal radial artery occlusion			
	The following day	One-month	3-month	6-month
1	22.22%(10/45)	20.00%(9/45)	17.78%(8/45)	20.00%(9/45)
2	28.57%(4/14)	21.43%(3/14)	14.29%(2/14)	14.29%(2/14)
3	33.33%(1/3)	33.33%(1/3)	33.33% (1/3)	33.33%(1/3)
4	100.00%(1/1)	100.00%(1/1)	100.00%(1/1)	100.00%(1/1)

d-RAO, distal radial artery occlusion; dTRA, distal transradial access.

### 3.4 Access site complications

No major complications (arterial dissection, pseudoaneurysm, or severe bleeding) were observed. Minor ecchymosis occurred in 5 cases (7.9%), resolving within 48 hours without intervention.

## 4 Discussion

HAIC, as an important interventional therapy for advanced HCC, can effectively control intrahepatic tumors, increase objective response rate, prolong overall survival, and improve quality of life. TFA, as the conventional approach of HAIC, has the remarkable limitation of restricting activities. This study demonstrates that dTRA is technically feasible for HAIC, with a 100% cannulation success rate and median puncture time of 3.5 minutes, comparable to reported TACE data (17, 20). The anatomical snuffbox provides a stable puncture site with direct access to the radial artery, avoiding the tortuosity often encountered in proximal TRA. The use of 4F sheaths and hydrophilic guidewires minimized vascular trauma, while ultrasound guidance improved first-pass success, particularly in patients with small artery diameters (mean 1.96 mm). These findings align with prior studies showing that dTRA puncture success rates exceed 85% with operator experience (21).

The absence of major ASC (e.g., bleeding, pseudoaneurysm) highlights the safety of dTRA in HAIC. This contrasts with TFA, which reports hematoma rates of 2–5% and pseudoaneurysm rates of 0.3–1.0% (22). However, the incidence of bruisin/hematoma during HAIC via TFA ranges from 9.6% to 18.2% (23, 24). The superficial location of the distal radial artery allows efficient manual compression, reducing hemostasis time to 3 hours—significantly shorter than the 6–12 hours required for TFA (25). Secondly, dTRA reduces bed rest, avoids femoral vein compression, eliminating the risk of DVT, a critical advantage in immobile HCC patients with coagulation abnormalities. Additionally, dTRA is particularly suitable for obese patients or those with groin pathologies, expanding the treatable population (12).

The observed d-RAO rate (25.4% early, 20.6% at 6 months) is higher than rates reported in coronary studies (5.7–13%) (13, 26), but lower than traditional wrist-level TRA (up to 30%) (27). This discrepancy may reflect prolonged procedure duration during HAIC, which often requires extended catheter placement (always 6 hours or 46 hours) and increases endothelial injury and thrombus formation, as well as the unique coagulopathy profile in cirrhotic HCC patients, who exhibit both hypocoagulable (low platelets) and hypercoagulable (elevated D-dimer) states, complicating hemostasis (28). Consistently, previous study revealed RAO for HAIC by TRA access occurred more frequently, up to 33%-37.5% (24), which is much higher than our results. Additionally, repeated punctures contribute to cumulative vascular damage, with ≥4 punctures showing a 100% occlusion rate in our cohort. Therefore, for patients requiring multiple HAIC sessions, strategies such as vascular protection or alternating bilateral radial artery use should be considered. Notably, d-RAO rates declined over time, possibly due to spontaneous recanalization or collateral development via the palmar arch. Early recanalization (within 3 months) was observed in 6.3% of cases, underscoring the importance of early Doppler ultrasound surveillance.

Preoperative D-dimer levels emerged as a significant predictor of d-RAO, reflecting systemic endothelial dysfunction and hypercoagulability in HCC (29). Elevated D-dimer may indicate microvascular injury, increasing susceptibility to thrombosis (18). Clinically, this suggests routine D-dimer screening to identify high-risk patients, who may benefit from intensified anticoagulation (e.g., higher heparin doses) or alternative access sites.

dTRA provides significant advantages for HCC patients, including improved comfort through early ambulation, preservation of femoral access for repeat interventions, and technical benefits such as easier catheter navigation via the left dTRA’s shorter path to the celiac trunk (30). Patient quality of life (QOL) measure was not provided in this study. Previous studied showed TRA to HAIC was associated with greater improvement in the quality of life associated with the procedure compared with TFA (24, 31). Furthermore, for patients with advanced liver malignancies undergoing HAIC treatment, TRA significantly outperformed TFA in terms of patient satisfaction and average hospital stay (23). Based on this, it can be inferred that HCC patients treated with the dTRA approach for HAIC may achieve higher comfort and better quality of life compared to those treated with the TFA approach.

However, its adoption is limited by factors such as the single-center retrospective nature of current study, no control groups, relatively small sample size and the need for longer-term patency data beyond six months. Future research should focus on prospective comparisons between dTRA and TFA for HAIC, as well as the development of vascular protection strategies like optimized antiplatelet regimens or drug-eluting sheaths. Additionally, refining patient selection through biomarker assessment—such as D-dimer and platelet function testing—could further enhance outcomes and minimize complications.

## 5 Conclusion

Distal transradial access for hepatic artery infusion chemotherapy is a feasible and safe alternative to traditional transfemoral access, with acceptable d-RAO rates and minimal major complications. Preoperative D-dimer screening and limitations on repeated punctures may optimize outcomes. However, large-scale prospective cohort studies are warranted to validate these findings and refine patient selection criteria.

## Data Availability

The raw data supporting the conclusions of this article will be made available by the authors, without undue reservation.
